# Doughnut-shaped bone lesions on ^18^F-FDG-PET/CT may indicate pseudoprogression under immuno(radio)therapy

**DOI:** 10.1007/s12149-026-02168-2

**Published:** 2026-02-12

**Authors:** Nathan D. Aeschbacher, Alexander Maurer, Stephan Beintner-Skawran, Michael Messerli, Bert-Ram Sah, Philipp A. Kaufmann, David Holzmann, Christian Meerwein, Lorenz Bankel, Tamara Rordorf, Nicolaus Andratschke, Panagiotis Balermpas, Matthias Guckenberger, Joanna Mangana, Daniela Mihic-Probst, Niels Rupp, Martin W. Huellner

**Affiliations:** 1 Department of Nuclear Medicine, University Hospital Zurich, Zurich, Switzerland; 2 University of Zurich, Zurich, Switzerland; 3 Department of Otorhinolaryngology, Head and Neck Surgery, University Hospital Zurich, Zurich, Switzerland; 4 Department of Medical Oncology, University Hospital Zurich, Zurich, Switzerland; 5 Department of Radiation Oncology, University Hospital Zurich, Zurich, Switzerland; 6 Faculty of Medicine, University of Zurich, Zurich, Switzerland; 7 Department of Dermatology, University Hospital Zurich, Zurich, Switzerland; 8 Department of Pathology and Molecular Pathology, University Hospital Zurich, Zurich, Switzerland

**Keywords:** Disease progression, Positron emission tomography computed tomography, Bone neoplasms, Lung neoplasms, Melanoma

## Abstract

**Purpose:**

Pseudoprogression (PP) is rarely observed in the skeleton. Over the past years, we have observed the appearance of unclassifiable, doughnut-shaped bone lesions in patients under immunotherapy. Our study aims to characterize these lesions under the hypothesis they may represent osseous PP.

**Methods:**

This retrospective study included 714 patients under immune-checkpoint inhibitor (ICI) therapy, targeted therapy, chemotherapy or local radiotherapy (RT) for either malignant melanoma (mM), lung cancer (LC), oropharyngeal or nasopharyngeal cancer with annular bone lesions, who underwent ^18^F-fluorodeoxyglucose positron emission tomography / computed tomography (^18^F-FDG-PET/CT) at the University Hospital Zurich between January 2010 and November 2023. Characteristics of annular bone lesions, including ^18^F-FDG-PET/CT metrics, were recorded. A descriptive analysis was carried out.

**Results:**

Six of the 714 patients demonstrated a new onset of ^18^F-FDG-avid doughnut-shaped bone lesions on ^18^F-FDG-PET/CT. Five patients were under ICI therapy and one patient was under RT. All lesions were transient and resolved on follow-up imaging. In two of six patients, histopathology samples were obtained and excluded malignancy. All ICI-treated patients demonstrated high ^18^F-FDG uptake of the lesions without corresponding abnormalities on computed tomography (CT). The mean time to lesion appearance after therapy initiation was 10.0 ± 8.1 months, and mean resolution occurred 7.7 ± 2.4 months after detection. All patients under ICI therapy exhibited additional immune-related changes.

**Conclusion:**

New, metabolically active doughnut-shaped bone lesions without clear CT correlates on ^18^F-FDG-PET/CT in patients receiving immunotherapy (or local RT) likely represent benign manifestations within the spectrum of PP rather than metastatic disease.

## Introduction

The introduction of immune checkpoint inhibitors (ICIs) has significantly improved overall survival (OS) and progression-free survival (PFS) in patients with various malignancies. While ICIs are increasingly applied in the adjuvant and neoadjuvant settings for locally resectable tumors, they are also broadely used in metastatic and incurable, locally advanced cancers, particularly in malignant melanoma (mM) and lung cancer (LC) [1–3]. During the past few years their indication has expanded to various other cancer entities such as advanced oropharyngeal and nasopharyngeal cancer [4, 5].

As a standard imaging modality for many types of cancer, ^18^F-fluorodeoxyglucose positron emission tomography / computed tomography (^18^F-FDG-PET/CT) enables the simultaneous assessment of disease on both metabolic and morphological levels. Because both malignant and inflammatory processes exhibit increased glycolysis and glucose uptake, resulting in high ^18^F-FDG avidity, accurate differentiation between these entities is essential for correct interpretation [6].

A counterintuitive phenomenon known as pseudoprogression (PP) has been observed following the initiation of immunotherapy, characterized by a transient increase in tumor burden before subsequent regression, or the transient appearance of new lesions. Differentiating PP from true progressive disease (TPD) remains a radiological and clinical challenge. The immune Response Evaluation Criteria in Solid Tumors (iRECIST, Table 1) criteria define PP as an initial increase in target lesion size followed by shrinkage or disappearance on follow-up imaging [7]. ^18^F-FDG-PET/CT specific response criteria have also been introduced (Table 1). The PET Response Criteria for Immunotherapy (PERCIMT) emphasize the number of new ^18^F-FDG-avid lesions as a predictor of treatment failure [8], while the immune PET Response Criteria in Solid Tumors (iPERCIST) incorporate both lesion number and metabolic activity [9]. Additionally, slightly adapted response criteria (such as immune-modified PERCIST 5 (imPERCIST 5)) have been proposed, offering further improvements in the prognostic performance of FDG-PET/CT through nuanced yet clinically meaningful refinements of the original iPERCIST framework [10]. However, all these frameworks rely on follow-up imaging, potentially delaying necessary treatment adjustments in patients with TPD. This underscores the ongoing need for reliable early markers to distinguish PP from TPD.

**Table 1:** Overview on different tumor response assessment criteria under ICI therapy. iRECIST, iPERCIST and PERCIMT in comparison. Table 1 adopted from Goldfarb et al. [9] (with kind permission from its publisher Springer Nature according to the creative commons attribution 4.0 international license (https://creativecommons.org/licenses/by/4.0/). Adaptations included replacing RECIST 1.1 and PERCIST criteria from the original table with PERCIMT criteria. PERCIMT criteria adopted from Anwar et al. [8] (with kind permission from its publisher Springer Nature, *License-Number 6121530597228)*. ^18^F-FDG-PET/CT, ^18^F-fluorodeoxyglucose positron emission tomography / computed tomography; SUV, standardized uptake value

	iRECIST	iPERCIST	PERCIMT
Complete Remission	Disappearance of all target and non target lesion nodes, regress to < 10 mm in short axis	Complete resolution of ^18^F-FDG uptake within the target lesion	No new lesions
Partial Remission	≥ 30% decrease in tumor burden compared to baseline (diameter in axial plane)	≥ 30% decrease in target tumor burden	No new lesions
Stable Disease	Neither partial, complete nor progressive disease	Neither partial, complete nor progressive disease	Neither partial, complete nor progressive disease
Progressive Disease	≥ 20% + 5 mm absolute increase in tumor burden. Appearance of new lesion or progression of non-target lesions. Need to be confirmed 4–8 weeks later; if progression is followed by tumor shrinkage, the bar is reset. Clinical stability is considered when deciding whether treatment is continued after immunologic undetermined progressive disease	≥ 30% increase in ^18^F-FDG lean body mass corrected SUV or advent of new ^18^F-FDG-avid lesions. Need to be confirmed by a second ^18^F-FDG-PET/CT at 4–8 weeks later; if progression is followed by partial mixed response (or stable metabolic disease), the bar is reset. Clinical stability is considered when deciding whether treatment ist continued after undetermined partial mixed response	≥ 4 new lesions of < 1.0 cm in functional diameter or ≥ 3 new lesions of > 1.0 cm in functional diameter or ≥ 2 new lesions of > 1.5 cm in functional diameter

PP occurs in up to 10% of patients with mM undergoing ICI therapy, but is significantly less frequent in LC patients, with an incidence below 5% and even more rare for patients with oropharyngeal or nasopharyngeal cancer (1.3%) [11–13]. In mM PP is more commonly associated with treatment using the cytotoxic T-lymphocyte associated protein 4 antibody ipilimumab compared to programmed cell death protein 1 inhibitors or programmed cell death protein 1 ligand inhibitors. PP is mostly observed around 12 weeks post treatment onset [13]. It tends to affect younger patients and is associated with improved OS compared to TPD [13]. However, PP might also be associated with a shorter PFS than that observed in patients with typical treatment response [13]. PP primarily affects visceral organs such as the kidneys and liver as well as the lungs, serosal surfaces (e.g., peritoneum) and lymph nodes [14]. PP in the skeletal system is also a recognized occurrence in solid tumors, particularly in the context of RT for pre-existing spinal lesions [15]. However, reports of new osseous lesions arising during immunotherapy are scarce. Among these few cases, some involved patients who received combined targeted therapy, while none had undergone prior RT [16, 17].

In recent years, we have observed the occasional emergence of unclassifiable, ring-shaped ^18^F-FDG-avid bone lesions on ^18^F-FDG-PET/CT in cancer patients. These patients underwent systemic therapy, typically with ICIs, either with or without radiotherapy. The aim of our study was to characterize these bone lesions on ^18^F-FDG-PET/CT and explore their potential relevance in the context of immunotherapy.

## Materials and methods

### Patients

This retrospective study received ethical approval from our institutional review board and was conducted in accordance with the International Council for Harmonization – Good Clinical Practice guidelines and the Declaration of Helsinki. Only patients with documented consent to contribute their data to retrospective research were included. The cancer entities included in this study were mM, LC, oropharyngeal and nasopharyngeal cancer.

**Fig. 1:**
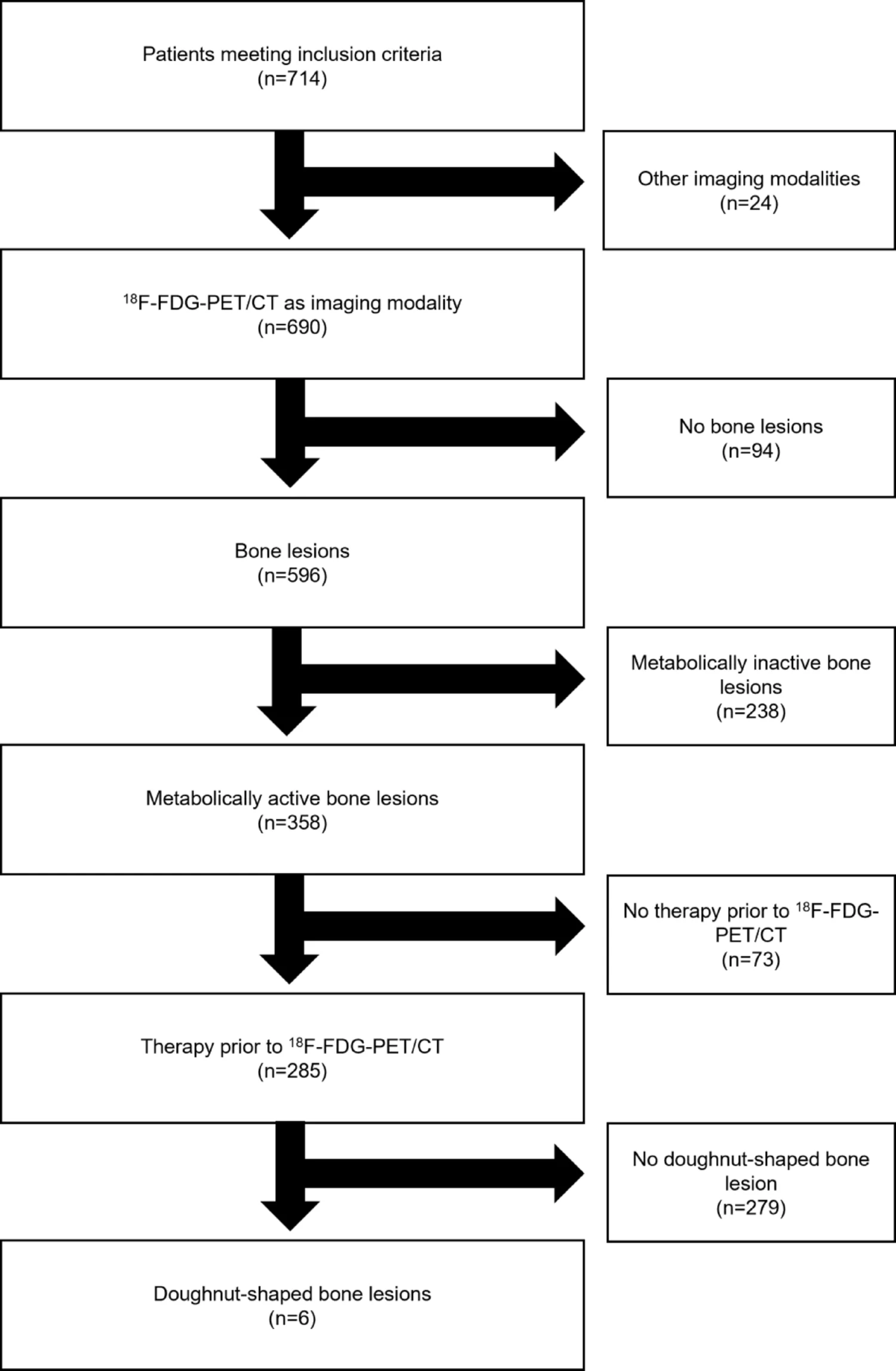
Flowchart of inclusion and exclusion criteria. ^18^F-FDG-PET/CT, ^18^F-fluorodeoxyglucose positron emission tomography / computed tomography

To identify eligible patients, a full-text search was performed in our radiology information system (Centricity™ RIS-I 7.0, GE HealthCare, Waukesha) for nuclear medicine studies conducted between November 2010 to November 2023, using keywords listed in Table 2. The respective tumor entities were selected because (1) they are comparably common in our population, (2) they are typically imaged with FDG-PET/CT, and (3) they are often treated using immunotherapy in our center. Based on these criteria, a total of 714 patients were retrieved and subsequently reviewed. After applying the exclusion criteria (Fig. 1), a total of six patients remained for further analysis, who displayed doughnut-shaped bone lesions.

**Table 2:** Keywords used for full-text search of patients in the radiological information system and retrieved studies. C10, ICD code for oropharyngeal cancer; C11, ICD code for nasopharyngeal cancer; C34, ICD code for lung cancer; C43, ICD code for malignant melanoma

Keywords	No. of patients
“C10” AND “bone metastasis” OR “bone metastases”	24
“C10” AND “osseous metastasis” OR “osseous metastases”	13
“C10” AND “ring-shaped”	0
“C11” AND “bone metastasis” OR “bone metastases”	38
“C11” AND osseous metastasis” OR “osseous metastases”	21
“C11” AND “ring-shaped	0
“C34” AND “bone metastasis” OR “bone metastases”	202
“C34” AND “osseous metastasis” OR “osseous metastases”	189
“C34” AND “ring-shaped”	6
“C43” AND “bone metastasis” OR “bone metastases”	131
“C43” AND “osseous metastasis” OR “osseous metastases”	83
“C43” AND “ring-shaped”	8

### ^18^F-fluorodeoxyglucose-PET/CT

All imaging was conducted at a single institution using standardized imaging protocols. The six patients were injected with a body weight adapted ^18^F-FDG dose (mean 192.8 ± 6.4 MBq). The scans were conducted on three different scanners from one vendor (Discovery MI™ -PET/CT in 5-ring or 6-ring configuration, and Discovery 710 PET/CT; GE HealthCare) and they were reconstructed as per standard protocols using ordered subset expectation maximation and/or block sequential regularized expectation maximization algorithms as documented in previous studies from our institution [18].

### Image Analysis

^18^F-FDG-PET/CT scans were reviewed by a triple board-certified nuclear medicine physician / radiologist / neuroradiologist with 17 years of experience. The lesions were analyzed and the following parameters were recorded: total lesion glycolysis (TLG), metabolic tumor volume (MTV), maximum standardized uptake value (SUV_max_), size of lesion, location and type of bone involved. Additionally, the type of immunotherapy, targeted therapy or RT received was documented, along with the time interval between the initiation of treatment and the ^18^F-FDG-PET/CT scan which showed these new bone lesions. Any further available data related to these lesions, such as histopathology findings (if available) and subsequent imaging findings, were also evaluated (Table 3 for further information).

**Table 3:** Patient characteristics. ^18^F-FDG-PET/CT, ^18^F-fluorodeoxyglucose positron emission tomography / computed tomography; PP, pseudoprogression; MTV, metabolic tumor volume; SUV_max ,_ maximum standardized uptake value; TLG, total lesion glycolysis; CR, complete remission; TPD, true progressive disease; PR, partial remission

Number of patient	Age (years)	Sex	Primary tumor	Tumor stage	Prior therapy	Time point of ^18^F-FDG-PET/CT after initiation of therapy (months)	Time until resolution of ring-shaped bone lesions (months)	Response at time point of ^18^F-FDG-PET/CT analyzed	Location of bone lesions	Cortical or medullar involvement	Number of lesions	Size of lesions (mm)	Histopath-ology available	MTV (cm^3^)	SUV_max_	TLG	Other organs with PP
1	53	F	Melanoma	III-A	Nivolumab	5	6	CR	T-spine 2 and 10	Medullar	2	25 × 19; 33 × 27	No	6.77; 11.1	4.71; 4.55	18679.6; 30828.6	Yes
2	54	F	Melanoma	III-A	Nivolumab	8	6	CR	T-spine 2 and 10	Medullar	2	29 × 23; 28 × 27	Yes	9.66; 12.08	4.39; 4.88	25491.8; 32435.4	Yes
3	55	F	Melanoma	IV-D	Nivolumab and ipilimumab; encorafenib and binimetinib	23/4*	10	New lymph node metastasis	T-spine 2 and 10	Medullar	2	29 × 22; 29 × 36	No	8.66; 17.82	8.56; 10.60	46205.7; 114926.1	Yes
4	73	F	Carcinoma of vestibulum nasi	III	Recent re-radiation and hyperthermic therapy. Proton beam therapy several years earlier	6	11	Local progression	Sphenoid sinus and clivus	Medullar and cortical	1	26 × 18	Yes	2.27	15.64	18582.5	No
5	49	M	Oropharynx cancer	IV-A	Nivolumab, radiation and chemotherapy	4	8	Local progression	Right acetabulum	Medullar	1	23 × 20	No	5.01	7.51	22868.5	Yes
6	72	F	Lung cancer	IV-B	Nivolumab, chemotherapy and radiation	3	5	Mixed response (TPD in brain, PR in lung, pleura and adrenal gland)	T-spine 5	Medullar	1	28 × 27	No	7.43	8.22	35556.6	Yes

*after addition of ipilimumab

### Data analysis

Due to the low number of patients (*n* = 6), we performed a descriptive analysis. With low patient numbers, statistical power decreases and the risk of false negative results (type II error) increases. Furthermore, subtle but statistically significant effects could be missed. In addition, results presented as significant could in fact be incorrect due to the small sample size [19, 20].

All calculations were carried out using Microsoft Excel™ (version 16.0.19029.20208, Redmond, WA).

## Results

**Fig. 2:**
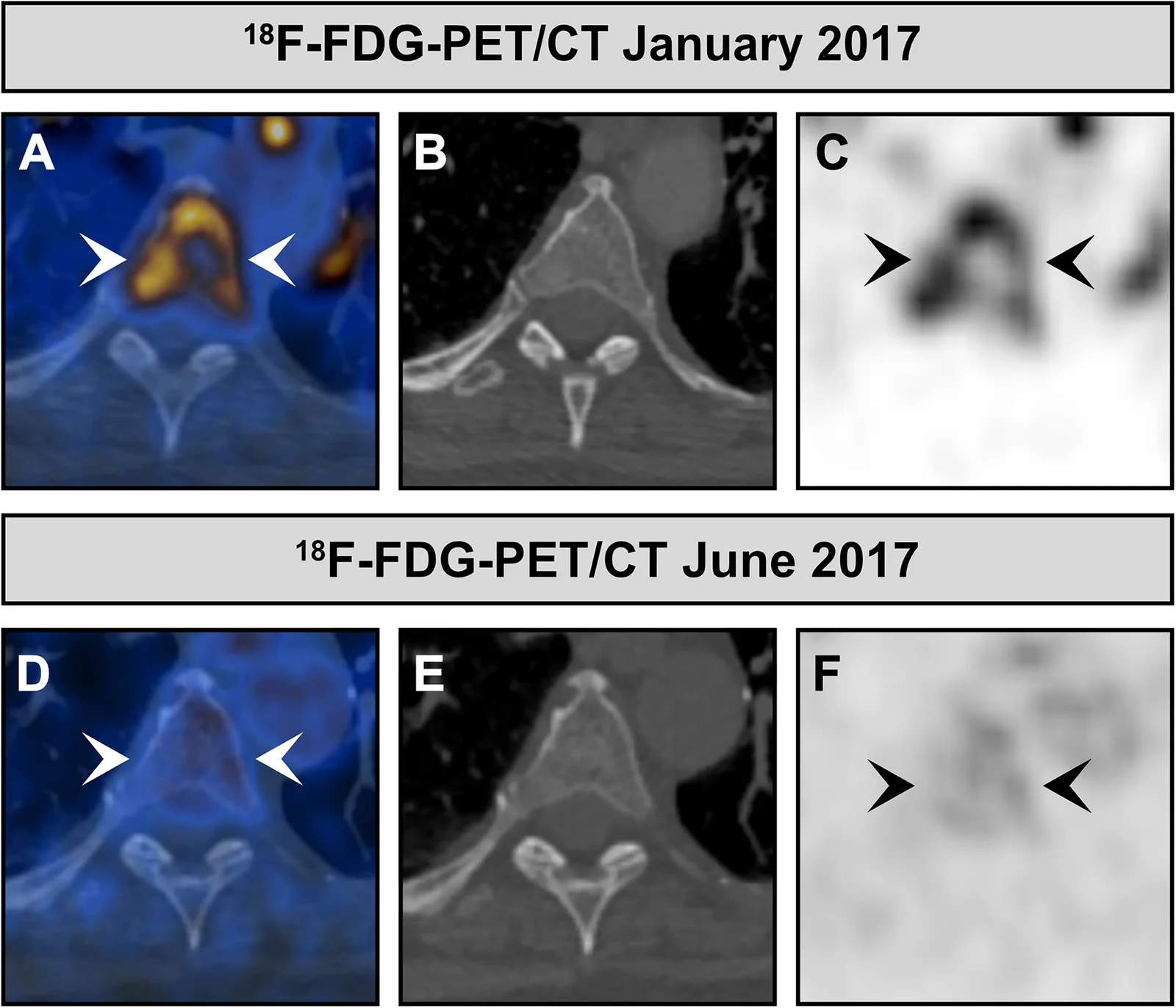
^18^F-fluorodeoxyglucose positron emission tomography / computed tomography (^18^F-FDG-PET/CT) images of patient no. 6. Metabolically active doughnut-shaped bone lesion (SUV_max_ 8.22, MTV 7.43 cm^3^, TLG 35556.6) in the 5th thoracic vertebral body (A, C) without cortical involvement. Resolution of the lesion was documented in a subsequent ^*18*^F-FDG-PET/CT scan 5 months later (D, F). Computed tomography shows no structural changes (B, E)

**Fig. 3:**
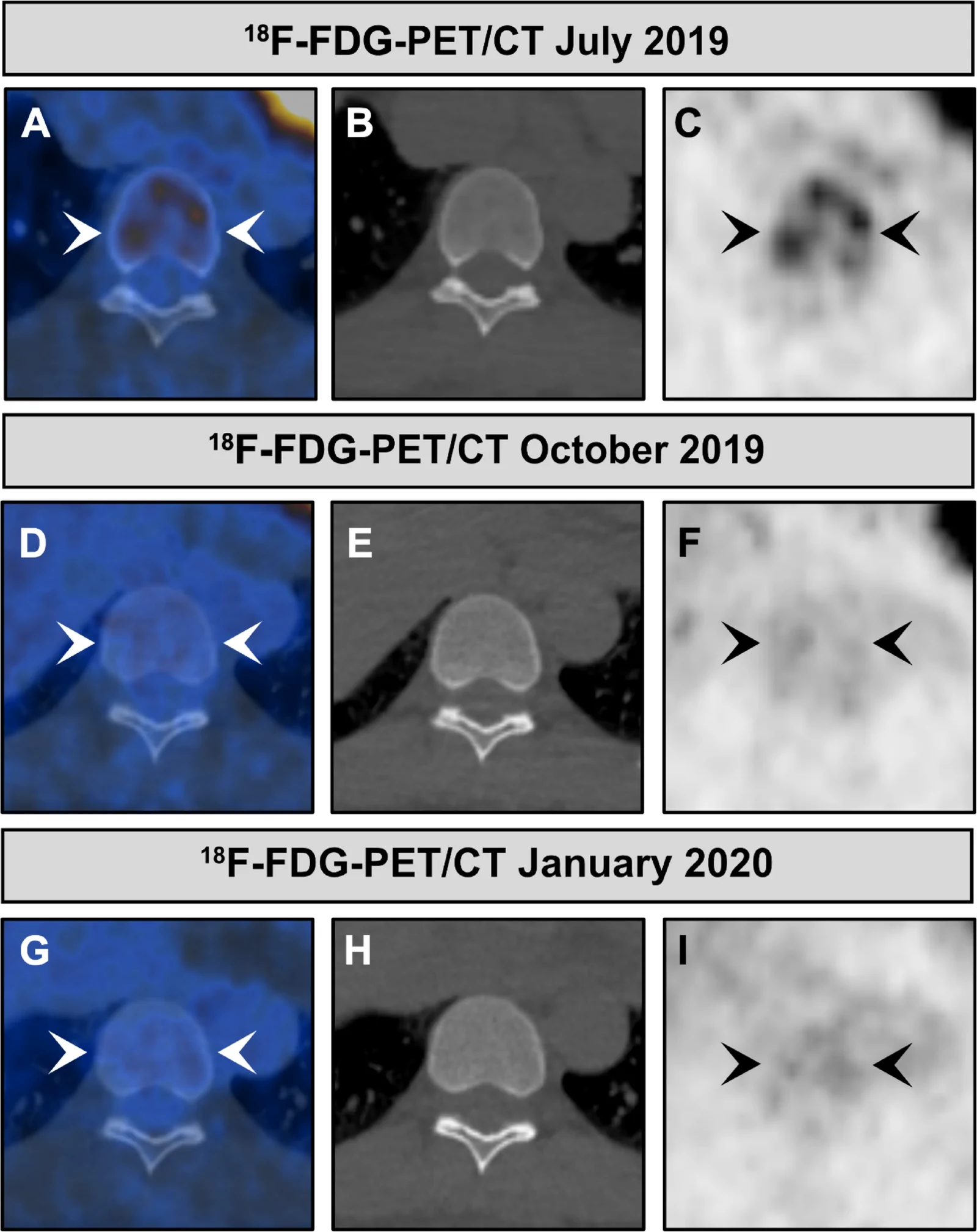
^18^F-fluorodeoxyglucose positron emission tomography / computed tomography (^18^F-FDG-PET/CT) images of patient no. 2. Metabolically active doughnut-shaped bone lesion (SUV_max_ 4.39, MTV 9.66cm^3^, TLG 25491.8) in the 10th thoracic vertebral body (A, C, D, F) without cortical involvement. Complete resolution was seen after 6 months (G, I). Computed tomography shows no structural changes (B, E, H)

A total of six patients were included for further analysis (Table 3), comprising nine ^18^F-FDG-avid bone lesions (mean 1.5 per patient; range 1–2). All lesions exhibited a characteristic doughnut-shaped morphology (Figs. 2 and 3). Five of the six patients were female, and the median age at lesion detection was 54.5 years (range 49–73 years). In three patients, the underlying cancer was classified as locally advanced by the multidisciplinary tumor board. Nivolumab monotherapy had been administered in four patients prior to imaging, one patient had received combined ipilimumab/nivolumab, and one patient was treated with RT and hyperthermic therapy in addition to proton beam therapy several years earlier.

The mean onset of doughnut-shaped lesions after initiation of ICI therapy was 8.6 ± 8.3 months. In contrast, in patient 4 - who had not received ICI therapy but was treated with RT and hyperthermic therapy (39.6 Gy over 1 month) - the lesions appeared after 17 months. Considering all patients, the overall mean onset time was 10.0 ± 8.1 months. The lesions resolved on average 7.7 ± 2.4 months after their initial detection. In five of the six patients, no corresponding structural bone changes were observed on CT during this interval, whereas patient 4 demonstrated subtle bone erosions.

The majority of these lesions (seven of nine) was located in the spine, with one lesion in the pelvic bones and one in the sphenoid bone/clivus. Lesions detected after ICI therapy were confined to the bone marrow without cortical involvement. In contrast, the skull lesion in patient 4, who had undergone RT, hyperthermic therapy and proton beam therapy several years earlier, also demonstrated subtle cortical changes.

In all ICI-treated patients, additional immune-related adverse events (irAEs) accompanied the appearance of annular bone lesions. All five patients exhibited lymphadenopathy, classified as sarcoid-like in three cases and reactive in two. One patient with reactive lymphadenopathy (patient 5) also developed other immune related adverse events (irAEs), including oligoarthritis and thyroiditis. Patient 6 showed additional subpleural nodules consistent with a sarcoid-like reaction.

The mean bone lesion size was 27.7 ± 2.8 mm by 24.3 ± 5.6 mm. The mean SUV_max_ was 8.3 ± 3.7 (ICI patients: 6.7 ± 2.4), mean bone lesion MTV was 9.6 ± 4.5 cm^3^ (range 2.3 to 17.8 cm^3^), and mean bone lesion TLG was 43’238.1 ± 30'015.9.

**Fig. 4:**
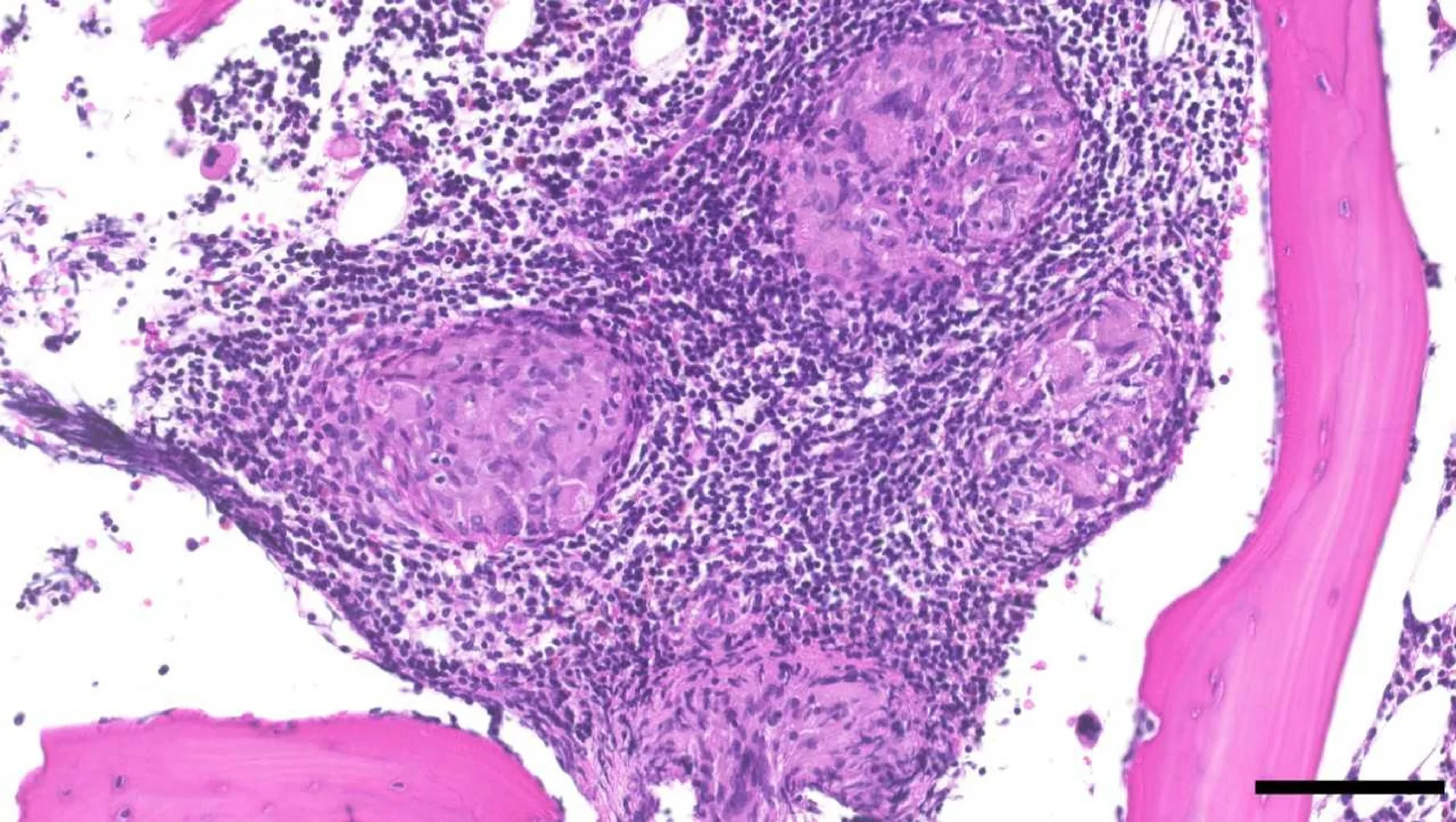
Histological section of bone marrow from the 10th thoracic vertebral body of patient no. 2 showing sarcoid-like, non-caseating granulomas composed of epithelioid histiocytes surrounded by reactive lymphocytes (H&E stain). Trabecular bone is visible on the right. Scale bar = 100 μm

In two cases, biopsies of annular bone lesions were performed. In patient 2 (after ICI treatment), histology revealed a granulomatous reaction with increased trilinear hematopoietic activity of the bone marrow consistent with sarcoid-like bone reaction (Fig. 4). In patient 4, who recently underwent RT and hyperthermic therapy as well as proton beam therapy several years earlier, biopsy showed inflammatory changes and metaplasia of the bone marrow. In both cases, no evidence of malignancy was found on histopathology.

At the time of ^18^F-FDG-PET/CT detection of the annular bone lesions, the state of the patients’ disease was as follows: patients 1 and 2 (33.3%) were in complete remission; patients 3, 4, and 5 (50%) had tumor progression requiring further therapy; and patient 6 (16.6%) demonstrated a mixed response , with progression of cerebral metastases but regression of pleural and pulmonary metastases.

## Discussion

Our study aimed to investigate transient, metabolically active, doughnut-shaped bone lesions detected on ^18^F-FDG-PET/CT. In a cohort of 714 patients with mM, LC, oropharyngeal and nasopharyngeal cancer, nine such lesions were identified in six patients. Five patients had received ICI therapy, while one had undergone local RT, hyperthermic therapy and proton beam therapy several years earlier. All five ICI patients demonstrated concomitant irAEs. Histopathology from two bone lesions in two different patients confirmed non-malignant findings. All lesions resolved on follow-up imaging. Primary tumor responses were heterogeneous: two patients achieved complete remission, three experienced tumor progression , and one showed a mixed response.

We hypothesize that newly emerging doughnut-shaped bone lesions represent transient, non-neoplastic changes within the spectrum of PP, such as sarcoid-like bone reaction, rather than metastatic disease.

Doughnut-shaped bone lesions have previously been described across imaging modalities, including ^99m^Technetium methylene disphosphonate (^99m^Tc-MDP) bone scintigraphy, X-ray, CT, and ^18^F-FDG-PET/CT. On ^99m^Tc-MDP bone scintigraphy, both giant cell tumors and avascular osteonecrosis have been reported with this morphology. As described by Levine et al., the photopenic center in giant cell tumors is not attributable to necrosis but to secondary aneurysmal bone cyst formation [21, 22]. In avascular osteonecrosis, the photopenic center reflects necrotic bone, whereas the peripheral radiotracer uptake corresponds to reactive bone formation or microcollapse [23]. This doughnut-shaped pattern has been reported at various anatomical sites, including the femur and skull [23–25].

Doughnut-shaped bone lesions are also characteristic of the familial calvarial doughnut lesions syndrome, or calvarial doughnut lesions–bone fragility syndrome, typically presenting as multiple, clinically asymptomatic skull lesions. In this entity, the ^99m^Tc-MDP-avid sclerotic rim corresponds to rapid structural changes, whereas the photopenic and radiolucent center reflects osteopenia [26]. Another reported case of a doughnut-shaped bone lesion involved a bone metastasis of a malignant peripheral nerve sheath tumor, which exhibited a doughnut-like configuration on CT but not on ^18^F-FDG-PET/CT, where the osteolytic regions also demonstrated high metabolic activity [27].

In contrast to the aforementioned conditions, all lesions under ICI therapy described in our study lacked clear morphological correlates on CT, suggesting a different underlying pathogenesis. Their transient appearance is reminiscent of PP, which is attributed to immune cell infiltration - particularly CD4^+^ and CD8^+^ T cells and macrophages [28] - resulting in tumor necrosis, edema, hemorrhage and ultimately transient lesion enlargement or a new and transient appearance of an hitherto occult metastasis after onset of ICI therapy [29]. IrAE, on the other hand, are believed to arise from autoinflammatory mechanisms. With the exception of necrosis, irAE and response to therapy (including PP) may have the same histological findings with an infiltration of CD4^+^ and CD8^+^ T cells and macrophages [30]. Importantly, irAE are clinically relevant and often necessitate therapeutic intervention or adjustment of ICI treatment, whereas PP is usually asymptomatic [31].

Our hypothesis that the observed doughnut-shaped bone lesions represent non-neoplastic changes within the spectrum of PP is supported by histopathology available in two patients, both showing absence of malignancy.

All five patients who received ICI therapy prior to imaging were treated with nivolumab, with one patient receiving combined ipilimumab and nivolumab therapy, in accordance with pertinent guidelines [32–34]. PP and irAE can occur with all ICIs, though the incidence varies by agent. While ipilimumab has a higher propensity to induce irAE, overall irAE are more frequently observed with nivolumab, the most commonly used ICI [29, 35].

We acknowledge that doughnut-shaped bone lesions may not be exclusively associated with ICI therapy. Patient no. 4, for instance, did not receive ICIs but underwent local re-RT and hyperthermic therapy after initial proton beam therapy years before. Histopathology revealed metaplastic squamous changes and inflammatory features, likely resulting from prior extensive treatment, as mentioned above. The transient, doughnut-shaped bone lesion was accompanied by presumed - though not histologically confirmed - osteoradionecrosis in the adjacent region of the sphenoid sinus and clivus. Accordingly, we hypothesize that the bone lesion in this patient reflects rather exogenous therapy-related effects with possible late transient osteoradionecrosis (a well-known possible late occurring toxicity after proton beam therapy [36]) than immunological mechanisms similar to ICI-related PP or irAE [29, 35].

In our study, doughnut-shaped bone lesions appeared on average 8.6 months after initiation of ICI therapy, later than typical PP or irAE in other organs, which usually occur within the first three months of treatment [13, 37]. However, onset varied considerably, with some lesions appearing up to 23 months after therapy initiation (patient 3). In this case, ipilimumab was added to nivolumab 4 months before the detection of the doughnut-shaped bone lesion. The observed comparably delayed onset may reflect a slower reaction of spongious osseous structures (apart from bone marrow), compared to endocrine organs or lymph nodes.

The developmental sequence of these bone lesions - whether they arise from focal precursor lesions or manifest directly as ring-shaped structures as a reaction to previously invisible metastatic lesions in the center of the ring - remains unclear, as ^18^F-FDG-PET/CT provides only a snapshot. In our patients, ^18^F-FDG-PET/CT was performed every three months to assess treatment response, as per institutional standards. It can therefore be assumed that the lesions developed at some point between two imaging sessions. Under the hypothesis of PP, one may speculate that cancer cells at the center of a lesion are initially recognized and eliminated by the immune system, sustaining a localized inflammatory response directed against tumor antigens. This response involves likely the recruitment of CD4+/CD8 + T cells and macrophages, forming granulomas similar to an osseous sarcoid-like reaction. Over time, this process could expand radially, giving rise to the characteristic doughnut-shaped appearance, akin to the well-known flare phenomenon [38]. To verify this hypothesis, imaging at shorter follow-up intervals and / or serial biopsies, and / or mapping biopsies would be required.

The bone lesions in our study resolved after a mean of 9.1 months, consistent with previously reported timelines for PP [39]. Their distribution and exclusive involvement of spongious bone with hematopoietic bone marrow without cortical changes may indicate ICI-induced immune activation. Currently, data on bone marrow-specific changes under ICI therapy are very limited. Prior studies have mainly addressed hematotoxic irAEs, which are rare; no evidence of bone marrow toxicity was observed in our cohort [40].

In some of the patients in our cohort, the number of new bone lesions in conjunction with their size would, according to PERCIMT criteria, be suggestive of progressive disease. Additional findings such as lymphadenopathy and other concurrent features in every patient further highlight the challenge of distinguishing true tumor progression from immunotherapy-related phenomena on imaging [8].

The median age at appearance of doughnut-shaped bone lesions was relatively young (54.5 years, range 49–73), compared to the median age of onset for lung cancer (70.9 years for women, 71.2 years for men) and melanoma (64.2 years for women, 69.1 years for men) in the Swiss population from 2017 to 2021 [41, 42]. Assuming these lesions represent a form of PP or bone-specific irAE, this observation is consistent with previous reports suggesting that younger patients exhibit a more active immune system, making them more susceptible to immune-mediated reactions [13, 39].

The SUV_max_ of the doughnut-shaped bone lesions (8.3 ± 3.7; ICI patients alone: 6.7 ± 2.4) was somewhat lower than typically observed in bone metastases from melanoma and lung cancer. However, as noted in previous studies, SUV_max_ alone is not a reliable discriminator between malignant and benign bone lesions [43]. This underscores the importance of assessing morphological changes on CT to differentiate metastatic disease from infectious or other non-neoplastic processes [44, 45].

Our study has several limitations. Its retrospective design and the small number of included bone lesions - within a population of patients with malignant disease under ICI therapy - result in limited statistical power, necessitating a descriptive analysis. The low patient numbers also increase the risk of overlooking subtle but clinically relevant differences between these lesions and metastases [19, 20]. Moreover, a single ^18^F-FDG-PET/CT represents only a snapshot, which is therefore not able to provide an exact timeline for appearance and resolution of the bone lesions. Finally, histopathological confirmation was available in only two out of six cases and did only include selected parts of the lesions.

In conclusion, newly appearing doughnut-shaped bone lesions on ^18^F-FDG-PET/CT in patients undergoing immunotherapy or local radiotherapy likely represent benign processes within the spectrum of PP, rather than metastatic disease. These lesions demonstrated significant ^18^F-FDG uptake without clear corresponding morphological changes on CT. Their transient appearance, often concurrent with irAE, supports the hypothesis of bone PP. However, based on the overall small number of patients with such doughnut-shaped bone lesions in our cohort, a generalization of this pattern as definitive indicator of PP is not possible. Further studies with larger patient cohorts and histopathological confirmation are warranted to clarify the underlying nature of these lesions and their potential as indicators of PP.
